# From Irritability to Amnesia: Unraveling Thalamic Glioma – a Case Report

**DOI:** 10.1192/bjo.2024.668

**Published:** 2024-08-01

**Authors:** Oyku Inanc, Tirth Dave

**Affiliations:** 1 Gulhane Training and Research Hospital, Ankara, Turkey; 2 Bukovinian State Medical University, Chernivtsi, Ukraine

## Abstract

**Aims:**

Gliomas, encompassing astrocytomas, oligodendrogliomas, and ependymomas, constitute the majority (40–55%) of primary brain tumors. Diagnosis can be challenging due to their uncommon nature, subtle symptoms, and diverse clinical manifestations. While neurological signs are typical, psychiatric symptoms may occasionally precede them. This case report explores a 59-year-old man whose initial psychiatric symptoms, resistant to treatment, evolved into memory impairment, ultimately revealing a high-grade glioma in the thalamus.

**Methods:**

A 59-year-old male patient presented to the psychiatric service, expressing concerns about excessive anger and aggression. His family observed his behavior as abnormal, noting uncharacteristic personality changes, particularly increased irritability. Following an outpatient psychiatric evaluation, he was diagnosed with excessive irritability. Over time, the patient's aggressive behaviors intensified, accompanied by feelings of being ignored and devalued by his family, heightened emotional sensitivity, and episodes of muteness. Despite two trials of medication (i.e., sertraline and alprazolam), there was a deterioration in adaptive functioning. Two years after the first onset, the patient experienced unfamiliarity with surroundings, forgetting place names, memories, and people's names. The patient had no family history of neurological or psychiatric illness, and there was no evidence of substance use in his past. To rule out organic causes, an MRI revealed a 17×21 mm lesion in the right thalamus and a calcified focus in the superior part of the left tentorium. Subsequent biopsy confirmed a high-grade glial tumor: anaplastic astrocytoma Grade III, with a Ki-67 index of 10%.

**Results:**

The extended onset of memory impairment in our patient, following a 3-year history of aggressive attacks and irritation, prompts an exploration of the intricate interplay between psychiatric and neurological manifestations. Unlike typical associations of personality changes with frontal lobe tumors, our case challenges this by implicating a thalamic tumor, highlighting the complexity of symptom correlation with precise brain lesion locations. Psychiatric symptoms, though not exclusive, may indicate underlying brain tumors. New-onset psychosis, mood or memory symptoms, atypical occurrences, personality changes, and anorexia in individuals over 40 warrant a thorough diagnostic workup, including neuroimaging, to investigate potential intracranial lesions.

**Conclusion:**

This case emphasizes the significance of identifying psychiatric symptoms as potential indicators of underlying brain tumors. The diverse manifestations, such as sudden psychosis, mood or memory changes, or unusual symptoms, should prompt further investigation, including neuroimaging. Early detection is crucial for improving overall quality of life, and understanding these psychiatric signs aids in unraveling the broader narrative of potential brain tumor involvement.

